# Towards an Effective Intrusion Detection Model Using Focal Loss Variational Autoencoder for Internet of Things (IoT)

**DOI:** 10.3390/s22155822

**Published:** 2022-08-04

**Authors:** Shapla Khanam, Ismail Ahmedy, Mohd Yamani Idna Idris, Mohamed Hisham Jaward

**Affiliations:** 1 Department of Computer System and Technology, Faculty of Computer Science and Information Technology, Universiti Malaya, Kuala Lumpur 50603, Malaysia; 2 Centre for Mobile Cloud Computing, Faculty of Computer Science and Information Technology, Universiti Malaya, Kuala Lumpur 50603, Malaysia; 3 School of Engineering, Monash University Malaysia, Bandar Sunway 47500, Malaysia

**Keywords:** Internet of Things, Variational AutoEncoder, Class-wise Focal Loss, data imbalance, intrusion detection, Deep Neural Network

## Abstract

As the range of security attacks increases across diverse network applications, intrusion detection systems are of central interest. Such detection systems are more crucial for the Internet of Things (IoT) due to the voluminous and sensitive data it produces. However, the real-world network produces imbalanced traffic including different and unknown attack types. Due to this imbalanced nature of network traffic, the traditional learning-based detection techniques suffer from lower overall detection performance, higher false-positive rate, and lower minority-class attack detection rates. To address the issue, we propose a novel deep generative-based model called Class-wise Focal Loss Variational AutoEncoder (CFLVAE) which overcomes the data imbalance problem by generating new samples for minority attack classes. Furthermore, we design an effective and cost-sensitive objective function called Class-wise Focal Loss (CFL) to train the traditional Variational AutoEncoder (VAE). The CFL objective function focuses on different minority class samples and scrutinizes high-level feature representation of observed data. This leads the VAE to generate more realistic, diverse, and quality intrusion data to create a well-balanced intrusion dataset. The balanced dataset results in improving the intrusion detection accuracy of learning-based classifiers. Therefore, a Deep Neural Network (DNN) classifier with a unique architecture is then trained using the balanced intrusion dataset to enhance the detection performance. Moreover, we utilize a challenging and highly imbalanced intrusion dataset called NSL-KDD to conduct an extensive experiment with the proposed model. The results demonstrate that the proposed CFLVAE with DNN (CFLVAE-DNN) model obtains promising performance in generating realistic new intrusion data samples and achieves superior intrusion detection performance. Additionally, the proposed CFLVAE-DNN model outperforms several state-of-the-art data generation and traditional intrusion detection methods. Specifically, the CFLVAE-DNN achieves 88.08% overall intrusion detection accuracy and 3.77% false positive rate. More significantly, it obtains the highest low-frequency attack detection rates for U2R (79.25%) and R2L (67.5%) against all the state-of-the-art algorithms.

## 1. Introduction

Due to the constant advancement and extensive demand of the Internet of Things (IoT), smart applications with advanced network technologies, big data, and devices that are connected to the internet have increased greatly. The application of IoT has already been witnessed in all walks of life [1]. However, due to the constrained nature of IoT on memory, processor, power, and information transmission, it suffers from significant security risks. Because several IoT nodes gather and store an enormous volume of users’ sensitive data, IoT has become an ultimate target for cyber adversaries [2]. For instance, a team from IBM X-Force Red carried out a security check on several smart city devices and discovered 17 security weaknesses in four cities [3]. Therefore, it is crucial to detect cyber-attacks on time to safeguard the network and its devices.

An Intrusion Detection System (IDS) is used to prevent and protect network devices from such security threats and vulnerabilities. Due to recent developments, IDS can identify and detect the attack types using Machine Learning (ML) and Deep Learning (DL) algorithms [4,5,6]. ML approaches include Support Vector Machine (SVM), K-Nearest Neighbour (KNN), Decision Tree (DT), Random Forest (RF) and Naïve Bayes (NB) [7,8,9], etc. and DL approaches include Deep Neural Network (DNN), Convolution Neural Network (CNN), Recurrent Neural Network (RNN), Long-Short term Memory (LSTM), AutoEncoder (AE), and Variational AutoEncoder (VAE) [10,11,12,13,14,15,16,17,18,19] etc. The efficiency of such learning methods have been investigated and verified using several publicly available datasets, such as KDD99, NSL-KDD, UNSWNB15, and Kyoto [20,21,22], and they achieved significant intrusion detection performance. For instance, the authors in [23] developed a DNN model with an integrated IoT architecture in order to maintain reliable and secure online monitoring for IoT vehicular applications. In another research work, a one dimensional CNN (1D-CNN) is proposed for fault diagnosis, which is robust against uncertainties and cyberattacks in IoT application [24]. Anomaly-based intrusion detection was investigated in order to secure IoT environments from cyberattacks in [25]. The authors in this article analyzed and reported the intrusion detection performance of existing deep learning techniques. These techniques achieved promising intrusion detection accuracy.

Notwithstanding the significant overall accuracy achieved by shallow ML and DL algorithms, IDS still suffers from a high False Positive Rate (FPR), inferior intrusion detection rates of low-frequency attacks because of the imbalanced nature of real-network datasets [26,27,28,29,30,31]. For instance, NSL-KDD [20] dataset contains five imbalanced classes. Several numbers of research have been proposed to develop data sampling or generation techniques to solve data imbalance problem [26,32,33,34,35,36]. The most common data oversampling algorithms that use the data-oriented approach are the Random Over Sampling (ROS), Synthetic Minority Oversampling Technique (SMOTE), Adaptive Synthetic Sampling Approach (ADASYN) [35,36,37]. Although ROS, SMOTE and ADASYN are classic methods for solving data imbalance issues, they are still popular among researchers [38,39,40,41,42]. Furthermore, some recent advancements in Variational AutoEncoder (VAE) [43], and Conditional VAE (CVAE) [44] algorithms are utilized to solve data imbalance issues by generating synthetic samples for minority classes. Nevertheless, these approaches highly depend on the cost sensitivity of learning algorithms. The cost matrix can be customized for better learning of misclassified samples using cost-sensitive learning. The represented cost matrix is used to reduce the probability of misclassification by many researchers [44,45,46,47].

However, the traditional classification methods still suffer from the following drawbacks. Due to the data imbalance problem, the majority class dominates the learning algorithms and the minority classes may not be learned effectively, hence leading to a high False Positive Rate (FPR), low minority-class attacks detection rate, and low overall detection accuracy. These issues could be solved by developing an appropriate data oversampling algorithm. Secondly, as conventional cross-entropy (*CE*) loss function is widely used as an objective function to train the oversampling algorithms, the majority class overwhelms the loss curve. This cannot enhance the quality and diversity of the synthesized minority class attack samples. Hence, the existing oversampling methods may not be able to improve the intrusion detection accuracy of the low-frequency attacks.

To overcome the above shortcomings and enhance high-quality data generation, we propose a novel IDS called Class-wise Focal Loss Variational AutoEncoder and Deep Neural Network (CFLVAE-DNN) intrusion detection model. Moreover, Focal Loss (FL) has emerged to enhance the power of *CE* as an alternative cost-sensitive learning to amplify the efficiency of learning algorithms [48,49,50,51]. We replaced the conventional reconstruction *CE* loss with the Class-wise Focal Loss (CFL) objective function to train the conventional VAE network. With the CFL loss function, we focus on the minority class samples more for a better representation of data for each class. In the learning process, the class-wise cost-sensitive approach aims to modify and re-weight the minority class samples. As a result, the VAE can generate minority class samples as close to the original input, which will, in turn, lead to better performance of intrusion classifier and reduce FPR, increase the detection performance of minority and low-frequency attacks. It is worth mentioning that the FL was implemented in intrusion detection very recently [46,47]. Additionally, several studies demonstrated that the FL improved the performance of deep learning algorithms in the field of computer vision and IoT applications [48,49,50,51].

Interestingly, the CFLVAE learns a better representation of minority class samples by utilizing the power of CFL and generates high-quality, diverse, and realistic synthetic samples to solve the data imbalance problem. The CFLVAE consists of an encoder, which compresses data into a lower dimension, and a decoder, which reconstructs the compressed distribution back to the original dimension. The generated data along with observed data is then passed to Deep Neural Networks (DNN), which serves as an intrusion detector to classify security attacks with lower FPR and higher detection performance. To sum up, the contributions of this research are highlighted as follows:

- A novel IDS based on Class-wise Focal Loss Variational AutoEncoder (CFLVAE) is proposed for data generation. A novel objective function called Class-wise Focal Loss (CFL) is designed for the proposed CFLVAE data generative model. The CFL objective function focuses on different minority class samples differently and learns the best distribution of observed data, which leads the CFLVAE to generate more realistic, diverse, and quality intrusion data.
- The Alpha (α) and Gamma (γ) parameters of the proposed CFL objective function are fine-tuned and optimized for individual minority class samples of the NSL-KDD intrusion detection dataset.
- A lightweight yet robust DNN model is developed to learn the features of high-dimensional balanced intrusion data to achieve high detection performance of low-frequency attacks.
- Finally, the proposed CFLVAE-DNN model is validated using the NSL-KDD dataset. Additionally, a comprehensive comparative study with relevant state-of-the-art learning-based IDS is provided.

The remainder of the paper is organized as follows. In Section 2, we review the related works on intrusion detection and stated the motivation of the work. The materials and methods of the proposed CFLVAE-DNN framework are described in Section 3 including derivative equations in detail. Section 4 presents experimental details. Experimental results and comparative studies are showcased in Section 5. Finally, Section 6 concludes the study and provides some future work.

## 2. Related Work and Motivation

A significant amount of research has been carried out towards innovative and efficient intrusion detection for IoT. Some of them utilized different conventional machine learning algorithms whereas others proposed deep learning methods. For instance, the authors in [6] proposed an AutoEncoder-based deep intrusion detection model named S-NDAE. Their model consists of two main parts: (1) Stacked Non-symmetric Deep AutoEncoders (S-NDAE) which is used for feature extraction and (2) trained S-NDAE and Random Forest (RF) are used for intrusion classification. The proposed S-NDAE experimented on NSL-KDD and KDD Cup’99 datasets. The model showed promising intrusion detection rates and achieved as high as 85.42% accuracy.

Ma et al. in [52] proposed a hybrid IDS called SCDNN. SCDNN uses Spectral Clustering (SC) to cluster the training and testing dataset into multiple subsets to train and evaluate the trained SCDNN model. Lopez-martin et al. reported an intrusion detection approach using Conditional VAE called ID-CVAE [53]. The proposed ID-CVAE is an encoder-decoder network and is based on unsupervised learning. ID-CVAE achieves 80.10% intrusion detection accuracy on the NSL-KDD dataset. Yin et al. proposed an RNN-based intrusion detection model called RNN-IDS [54]. They experimented with different hyper-parameters such as learning rates and the number of hidden nodes to obtain optimal training time and detection accuracy. The model was evaluated using KDDTest+ and KDDTest-21 dataset [55] and obtained 83.28% and 68.55% accuracy respectively.

Li et al. [56] experimented on a different number of hidden layers on LSTM and Gated Recurrent Unit (GRU) based deep RNNs approach. The model consists of an extended learning system to perform intrusion classification. The experiments on two benchmark datasets namely NSL-KDD and BGP showed the significance of hidden layers in detection accuracy for the proposed neural network. Interestingly, the model obtained significant detection accuracy and F1-score.

The authors, Vinayakumar et al. in [5], proposed a scale-hybrid-IDS-AlertNet (SHIA) model based on deep neural networks to monitor network traffic. The proposed system can identify the malicious events for both network and host levels to further alert network administrators. Likewise, the SHIA model was evaluated on multiple intrusion datasets and performed better than state-of-the-art machine learning models.

The majority of network traffic in a real environment is uneven, which means the attack traffic is considerably lower compared to normal network traffic. This leads to a class imbalance problem which degrades classification accuracy and escalates the FPR of the learning model. Some recent research has focused on addressing the data imbalance problem to improve detection accuracy. Many popular oversampling methods exist such as ROS [37], SMOTE [36], ADASYN [35], Generative Adversarial Network (GAN) [57,58], AutoEncoder (AE) [59] to solve data/class imbalance problem.

The authors in [60] explored the significance of Conditional Variational AutoEncoder (CVAE) to generate data and solve data imbalanced issues to improve intrusion classification. An improved version of CVAE (ICVAE) is used to generate new data samples and DNN is utilized for classifying intrusion in the system. The ICVAE-DNN model outperforms in detecting minority attack categories. However, they may neglect the cost sensitivity of imbalance intrusion data to generate high-quality synthetic data. The traditional *CE* loss in ICVAE may not be able to optimize the latent distribution and may lead to degrading the quality of decoded samples. Therefore, the generated data deviate greatly from observed data, which leads the classifier to perform poorly.

Although the aforementioned intrusion detection approaches including data generation methods succeeded with satisfactory performance, they yet suffer from inferior detection rates, high FPR, and low detection performance of low-frequent, minority, and unknown attack classes.

To overcome these issues, this work proposes a novel intrusion detection framework called CFLVAE-DNN. To better apprehend the representation and attributes of the observed intrusion samples and their minority attack samples, we design a novel objective function called CFL inspired by Focal Loss (FL) for the proposed CFLVAE data generative model. CFLVAE-DNN inherits the strengths of Variational AutoEncoder (VAE) and utilizes Class-wise Focal Loss (CFL) as an objective function instead of the traditional *CE* to train the CFLVAE model. Moreover, the model focuses on the minority class samples and adjusts weights for each class sample individually. CFLVAE-DNN framework consists of two phases: (1) CFLVAE is trained to generate realistic synthetic data, (2) the DNN classifier is used for classifying the attack categories.

## 3. Materials and Methods

The proposed CFLVAE-DNN model inherits the property of Variational AutoEncoder (VAE) for data generation. VAE is improved by adding Class-wise Focal Loss (CFL) as an objective function. The CFL objective function assigns different weight properties to the different target classes, hence, this leads to generating high quality, diverse and realistic data for minority class attacks. The following sections explain the VAE and how the proposed CFL is incorporated with VAE.

### 3.1. Variational AutoEncoder (VAE)

**Figure 1:**
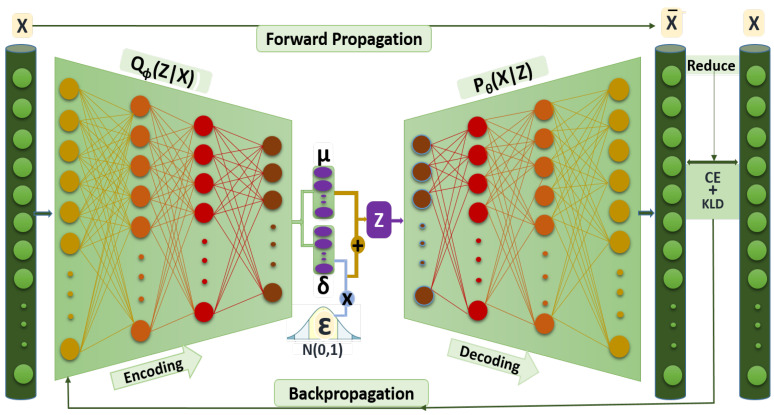
Variational AutoEncoder with CE loss.

Variational AutoEncoders (VAE) is a variation of AutoEncoder (AE), which can generate synthetic data [6]. Traditionally, VAE architecture consists of an encoder $Q_{\phi }\left(Z|X\right)$, a latent space Z and a decoder $P_{\theta }\left(X|Z\right)$ [43,60]. The architecture is based on the encoder-latent space-decoder paradigm. The latent space of VAE is a distribution with mean and variance. Figure 1. depicts the VAE architecture with traditional cross-entropy (*CE*) loss function.

In VAE, the encoder conventionally transforms the input data to a lower dimension with a probability distribution. Moreover, for the latent space *Z* to have a meaningful abstract property to reconstruct the observed data, the distribution is regularized, and VAE learns variational inference during the training. The encoder network’s weight parameter ϕ is learned to encode the input samples to produce encoded feature representation *Z*. In contrast, the decoder network’s weight parameter θ is trained to reproduce new samples by mapping the encoded space *Z*. During the training process, some information can be lost and may not be recovered while decoding. The main drive is to obtain the best encoder-decoder pair that ensures maximum information gain during encoding and has minimum reconstruction error during decoding.

VAE model is widely used to generate data by passing sampled *Z* to the decoder. During the forward propagation, the reconstruction error (e.g., *CE* loss) and *Kullback–Leibler* (*KL*) divergence loss, $D_{KL}\left[Q\left(Z|X\right)||P\left(Z\right)\right]$ can be computed, and the network back-propagates the computed error value. Hence, the lower bound loss function of VAE is expressed as the equation below [15,43]:

(1) $$L_{vae}\left(\phi ,\theta ,X\right)=E\left[\log\left(X|Z\right)\right]-D_{KL}\left[Q\left(Z|X\right)||P\left(Z\right)\right]$$

where, E[log(X|Z)] is the reconstruction loss which is traditionally cross-entropy (*CE*) loss used in VAE. Hence, the variation lower bound of VAE can be re-written as:

(2) $$L_{vae}\left(\phi ,\theta ,X\right)=-log\left(p_{t}\right)-D_{KL}[Q\left(Z|X\right)||P\left(Z\right)]$$

The first part ($-log(p_{t})$) is the *CE* loss and second part is the *KL* divergence loss. The *CE* loss will further be elaborated in the next section.

### 3.2. Proposed Class-Wise Focal Loss Variational AutoEncoder (CFLVAE)

We aim to reconstruct data for a specific minority class and hence, we will train our VAE model by adding sample data with the class label *y*. The encoder of VAE can then be expressed as $Q_{\phi }\left(Z|X,y\right)$ and decoder as $P_{\theta }\left(X|Z,y\right)$ [44,61]. During training, the network learns to encode the best latent distribution *Z* for specific class label *y*. The joint vector of *Z* and *y* is then passed to the decoder to reconstruct a new attack vector *X* for specified class label *y*. The loss function of the VAE is computed using the following equation [44]:

(3) $$L_{vae}\left(\phi ,\theta ,X,y\right)=-log\left(p_{t}\right)-D_{KL}[Q\left(Z|X,y\right)||P\left(Z|y\right)]$$

where $L_{cvae}\left(\phi ,\theta ,X,y\right)$ is the variation lower bound of VAE. The first term is the typical cross-entropy (*CE*) loss [48,62] and is defined as follows:

(4) $$CE\left(p_{t}\right)=-log\left(p_{t}\right)$$

**Figure 2:**
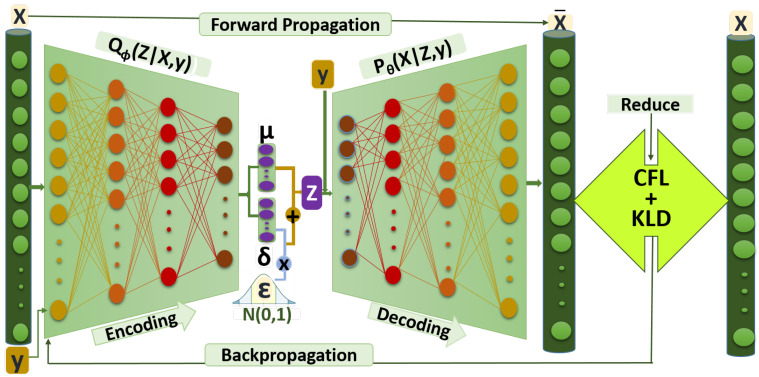
Class-wise Focal Loss Variational AutoEncoder (CFLVAE).

We replaced the conventional *CE* loss of VAE with Class-wise Focal Loss (CFL), which we termed CFLVAE. The architecture of CFLVAE is shown in Figure 2. As mentioned above, the traditional *CE* loss in VAE may not be able to optimize the latent distribution. By using *CE* as reconstruction loss the majority class in an imbalanced dataset dominates the loss and governs the gradient. On the other hand, the CFL loss function focuses on the minority class and adjusts weights for each class sample individually. This allows the VAE to generate realistic and diverse data to solve the data imbalance problem for intrusion detection.

We added a modulating factor $\left(1-p_{t}\right)$ with tune-able parameter γ to overcome the issues with *CE* loss, which is called FL loss [48]. $\left(1-p_{t}\right)$ is used to take into consideration the hard/misclassified and easy/true negative samples. Formally, the mathematical expression of FL [48] is as follows:

(5) $$FL\left(p_{t}\right)=-\alpha_{t}{\left(1-p_{t}\right)}^{\gamma }\log\left(p_{t}\right)$$

where, α term is added to handle the class imbalance problem where,

(6) $$\alpha_{t}=\left\{\begin{array}{c} -\alpha ,y=1\\ -\left(1-\alpha \right),\mathrm{otherwise} \end{array}\right.$$

$\alpha_{t}$ is a weighted term whose value is α for positive class and 1−α for negative class. The term α balances the significance of majority/minority examples.

This study considers different values of γ>0 for different classes depending on their imbalance nature to minimize the relative errors for minority classes by paying more attention to them. The hyper-parameter γ regulates the nature of the loss curve. A larger value of γ leads to a lower loss for minority class samples. We considered several values of γϵ[0,10] shown in Table 1. The focusing parameter γ smoothly adjusts the rate at which easy examples are down-weighted.

The idea behind the FL is to minimize error input from well-recognized examples and maximize the error value for the examples which accept a low loss. Hence, the final loss equation of CFLVAE is formulated as below:

(7) $$L_{cflvae}\left(\phi ,\theta ,X,y\right)=-\alpha_{t}{\left(1-p_{t}\right)}^{\gamma }\log\left(p_{t}\right)-D_{KL}[Q\left(z|X,y\right)||P\left(z|y\right)]$$

The first term is the CFL loss $\left(-\alpha_{t}{\left(1-p_{t}\right)}^{\gamma }\log\left(p_{t}\right)\right)$, which is the reconstruction loss of our proposed CFLVAE.

FL loss is used for cost-sensitive learning to stabilize cross-entropy loss, so that the rare examples are learned efficiently. The adeptness of FL has been applied and tested for object detection, computer vision in an imbalanced dataset, and attained incredible performance [48]. However, the usefulness of FL is not restricted to only computer vision; it is also applied to intrusion detection for imbalanced data issues [46].

### 3.3. Proposed Intrusion Detection Framework

**Figure 3:**
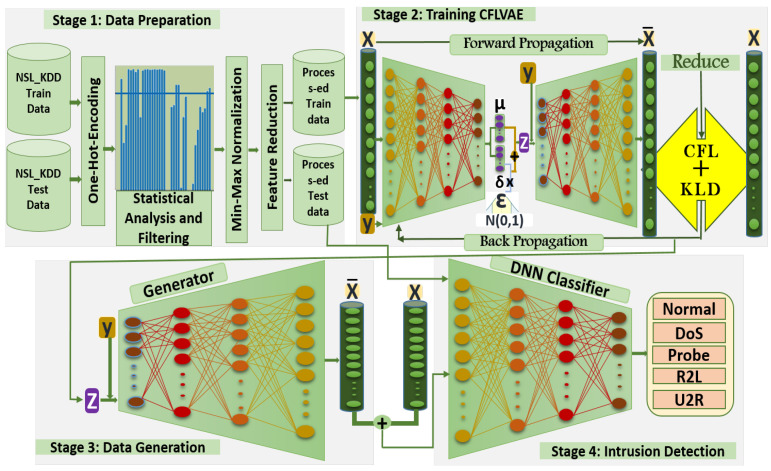
Proposed CFLVAE-DNN Framework.

The framework of the proposed CFLVAE-DNN is presented in Figure 3. CFLVAE-DNN mainly comprises four stages: (1) Data preparation: firstly, discrete features are converted to numeric values. Secondly, the features with mostly zeros are eliminated. Finally, data are normalized between 0 and 1. (2) Training CFLVAE: Class-wise FL is added to VAE for cost-sensitive learning to better model the minority class intrusion data. The model is trained to learn a better representation of minority class samples. (3) Data generation: generating realistic and diverse synthetic samples for specified minority classes using trained CFLVAE and balancing the dataset. (4) Intrusion detection: using the balanced dataset to train the DNN classifier to classify intrusions effectively.

#### 3.3.1. Data Preparation

As mentioned above, the first stage of CFLVAE-DNN is to preprocess the data. NSL-KDD dataset is preprocessed using the following steps using Algorithm 1.

Feature numeration: One-Hot encoding [63] is one of the most simple, effective, and widely used techniques to convert categorical or discrete features to numerical features. It transforms the categorical values to binary vectors with 0 s and 1 s. 1 corresponds to the existence of a particular categorical value. In NSL-KDD dataset, there are three discrete features such as protocol type, service, and flag. We utilized the strength of One-Hot encoding to convert all discrete values to numeric values.

Feature filtering: We eliminated all irrelevant features. The ratio of zeros is computed for each numerical feature and the features with more than 90% of zero value are removed. Stage one in Figure 3 depicts the percentage of zeros of each feature in the KDDTrain+ dataset which has been eliminated.

Data normalization: It is important to scale the values to a certain range for the deep learning models to be trained efficiently. NSL-KDD datasets include values with dynamic range. The linear conversion of the original input, all feature values are scaled to the range [0–1] using min-max normalization [64] as the following equation:

(8) $$x^{{}^{\prime }}=\frac{x-min(x)}{max(x)-min(x)}$$

where, $x^{{}^{\prime }}$ is the normalized value and *x* is the original value of a dataset.

Feature reduction: To reduce the computational complexity of the model for resource-constrained IoT devices and to maximize the performance of the classifier, a common feature selection approach, called Mutual Information (MI) has been utilized for feature selection on the basis of the information value. According to the authors [65,66], The MI between two random variables *X* and *Y* can be is defined as:

(9) MI(X;Y)=H(X)−H(X|Y)

where, MI(X;Y) is the mutual information value for variable *X* and *Y*, H(X) denotes the entropy for variable *X* and H(X|Y) denotes the conditional entropy for *X* given *Y*. The output is denoted as the units of bits. MI is a estimation of mutual dependency between two random variables. As such, the measure is symmetrical, meaning that MI(X;Y)=MI(Y;X). The final 87 features are selected to train both CFLVAE and DNN networks.
**Algorithm 1:** Data Preparation.**Input**: Imbalanced raw dataset**Output**: Pre-processed dataset1   *Function:*2     *Numeration* ← One-Hot-Encoding to convert discrete data to numeric data3     *Feature filtering* ← filter out unimportant and redundant features with 90% of zeros4     *Normalization* ← perform min-max normalization to scale data between (0, 1)5     *Feature reduction* ← utilize MI feature reduction technique to select best features6     **Return** scaled dataset with expanded and important features7   *End of the Function*

#### 3.3.2. Training CFLVAE

The second stage of the CFLVAE-DNN model is to train the CFLVAE data generation model. CFLVAE training consists of the following steps based on Algorithm 2. The encoder is trained to obtain the best distribution of latent code Z and the decoder is trained to recreate the data from learned latent distribution Z. We train the proposed CFLVAE network to reduce the KL divergence loss and the Class-wise Focal loss (CFL). KL divergence is used to minimize the distance between the reconstructed samples $\bar{X}$ and the observed samples *X*, that is, to decrease the $D_{K}$*L* loss to recreate data from the Multivariate Gaussian prior P(z). CFL is used to minimize the reconstruction error by learning weights for each class. The training procedure is done in a number of mini-batches and epochs for the weight parameters ϕ and θ of the CFLVAE networks to be converged effectively. We utilized ReLU6 [67,68] as activation function and Adam optimizer [69] to train the CFLVAE generation model.
**Algorithm 2:** CFLVAE for generating synthetic data samples.
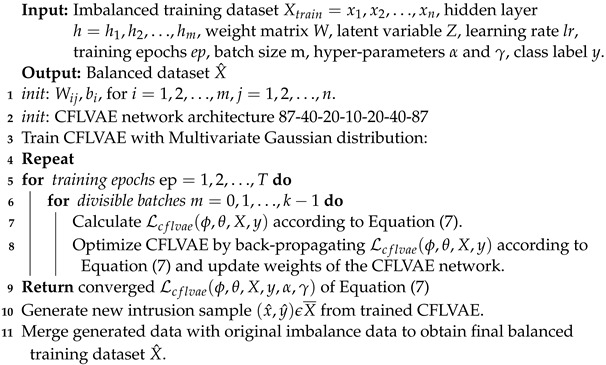


#### 3.3.3. Data Generation

In this experiment, we make use of a random sampling method to sample data points from the trained CFLVAE. Firstly, we concatenate the minority class label *y* with data points from *Z* and feed them to the decoder network. In other words, after training the CFLVAE with CFL loss, we pass the encoded *Z* distribution to the decoder along with its respective class level *y* to generate the desired number of synthetic samples. Then, in the encoder network, standard normal distribution $Q_{\phi }\left(Z|X,y\right)$ is used to obtain latent space *Z*. Afterwards, a point from *Z* is then passed to the decoder $P_{\theta }\left(X|Z,y\right)$, added with standard normal distribution N(0,I) for respective minority class label *y* to augment a new training sample $\left(\hat{x},\hat{y}\right)$. Meanwhile, we assure that the generated training example corresponds to a specific minority attack class *y*.

#### 3.3.4. Intrusion Detection

In this work, Deep Neural Network (DNN) model is utilized as a classifier with a customized architecture for intrusion detection. The DNN is a neural network model comprised of one input, one output, and several hidden layers [70]. Moreover, our proposed DNN model is a fully connected feedforward neural network. Apart from the input and output layers, our proposed DNN architecture consists of three hidden layers. Furthermore, we utilized ReLU6 [67,68] as the activation function of all hidden layers and softmax for the output layer. As the input dimension of the classifier is the same as the CFLVAE networks, our DNN model is expected to perform well.

Indeed, the DNN classifier can extract the most relevant attributes automatically. The weight initialization of the classifier is done in the same way as CFLVAE networks. The generated minority class samples merged with observed samples are fed into DNN to train the classifier. The most frequently implemented loss function for multi-class classification task is the categorical cross-entropy ($CE_{c}$) loss function [71]. Hence, for our proposed DNN classifier, the $CE_{c}$ loss function is defined as follows:

(10) $$CE_{c}=\sum_{i=1}^{n}y_{i}\cdot \log\hat{y_{i}}\;$$

where, $\hat{y}$ is the predicted class label.

Additionally, in finding the optimal network architecture, this research implemented different network architectures by changing the number of hidden layers from six(6) layers to one(1) layer. The optimal network architecture is important to achieve the optimal detection accuracy as well as to fit the model into resource-constrained IoT devices. The selected model should be as light as possible.

We utilized a bias regularizer with a value of 0.0005 and the learning is optimized by the Adam optimization algorithm [69]. To evaluate the classifier we fed the NSL-KDDTest+ and NSL-KDDTest-21 data into trained DNN to obtain intrusion detection performance. The proposed DNN classifier is elaborated in Algorithm 3.
**Algorithm 3:** DNN Classifier.
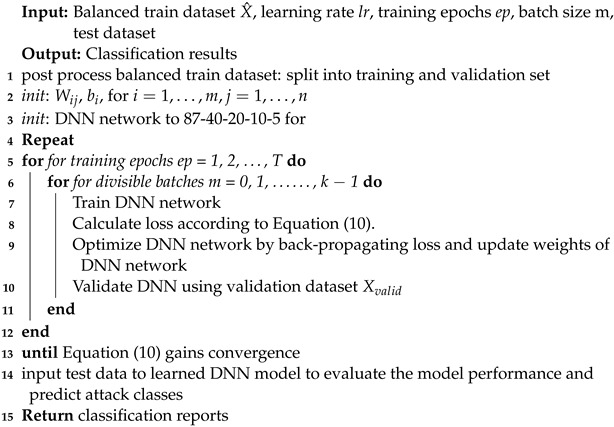


### 3.4. Performance Matrix

For an effective evaluation of our proposed IDS, we have considered the seven most widely used performance metrics including accuracy, precision, recall, F1-score, False Positive Rate (FPR), and Receiver Operating Characteristic (ROC). Area Under the ROC Curve (AUC) is also measured to evaluate the performance of our proposed model. The parameters are mainly obtained out of the confusion matrix of detection algorithms [72].

Likewise, the confusion matrix is formed based on the true positive (tp), true negative (tn), false positive (fp), and false negative (fn) matrix. Correctly predicted traffic is called tp, meanwhile, tn is the number of benign network traffic, which is correctly classified, fp is the number of misclassified traffic and finally, fn the number of traffic incorrectly predicted as benign traffic. Indeed, the higher the accuracy, precision, recall, and F1-score, the better the performance of the intrusion detection algorithm. Similarly, the lower value of the FPR is expected for better performance of the detection algorithm.

Accuracy is defined as the ratio of the number of accurately classified attacks and benign traffic to the total traffic. Accuracy is mathematically expressed as follows:

(11) $$\mathrm{Accuracy}=\frac{tp+tn}{tp+tn+fp+fn}$$

The Recall or Detection Date (DR) is defined as the percentage of correctly predicted actual attacks. The recall is also known as sensitivity or True Positive Rate (TPR). The mathematical expression of DR is as follows:

(12) $$\mathrm{DR}/\mathrm{Recall}=\frac{tp}{tp+fn}$$

Precision is the probability of all classified attack traffic, which are true attack traffic. Precision is can be expressed as below:

(13) $$\mathrm{Precision}=\frac{tp}{tp+fp}$$

F1-score is computed as the harmonic averages of accuracy and DR. F1-score is used to observe the overall performance of the IDS. The equation of F1-score is defined as:

(14) $$F1-\mathrm{score}=\frac{tp}{tp+fp+fn}$$

FPR is the measure of the probability of incorrectly predicted benign data traffic. The equation of FPR is expressed as:

(15) $$\mathrm{FPR}=\frac{fp}{tn+fp}$$

ROC is a two-dimensional curve of FPR and TPR with possible thresholds for the transition of observation to a particular target variable. The AUC refers to the area under the ROC curve. The ideal value of AUC is between 0.5 and 1 for a good classifier. AUC is expressed as:

(16) $$\mathrm{AUC}=\int_{0}^{1}\frac{tp}{tp+fn}d\frac{fp}{tn+fp}$$

## 4. Experiments

In evaluating the proposed CFLVAE-DNN model, we have selected a highly imbalanced NSK-KDD intrusion dataset. In this section, the details about the benchmark dataset, its preprocessing steps, and the implementation details are presented.

### 4.1. Benchmark Imbalanced Dataset

Many recent studies relied on the well-known NSL-KDD dataset [55] to validate Network IDS (NIDS) and its ML algorithms. NSL-KDD is a highly imbalanced network intrusion dataset. The class imbalance of this dataset is shown in Figure 5a in Section 5.1. Interestingly, the dataset comprises four attack vectors (DoS, Probe, R2L, U2R) and normal network traffic. However, the total attack techniques are not limited to these four.

Moreover, NSL-KDD has a variety of useful information to detect and mark malicious network traffic. Some of the important features comprise the ability to extract data from the packet header, thereby, uncovering the required information. Its content features carry the information about the actual payloads. Namely, time-dependent features enable the study of the traffic request over two seconds. Accordingly, the host-based features access the dynamic behavior over a sequence of active connections. The IPv4&6, TCP, and UDP are widely used protocols in Wireless Sensor Networks (WSNs), whereas FTP, SNMP, ARP, and XTerm are uncommon in WSN environments. Furthermore, few attacks are created for Windows and Linux Operating Systems only. More precisely, DoS and Probe attacks are interesting to be tested in resource-constrained environments.

NSL-KDD dataset is an upgraded version of the KDD-99 [20], aimed to address the redundant records problem of the earlier. The NSL-KDD dataset comprises 125,973 samples in total and there are 25,192 (20%) training samples and 22,544 (KDDTest+) and 11,850 (KDDTest-21) test samples. In this study, we utilized 25192 (20%) training samples to train and both test datasets to evaluate our model. NSL-KDD dataset has 41 features: 38 continuous and 3 categorical (discrete values). This study has performed additional data transformation as well. Given the skewness of several categories of attack classes in the NSL-KDD dataset, it is harder to assess categories by just using original class labels. Some intrusion vectors only exist in the test dataset but not in the training dataset, which makes the classifier perform inefficiently. The following section defines the DoS, Probe, R2L, and U2R attacks in detail:

Denial of Service (DoS)—the invader exhausts available computational power or memory space making the system full victim of resource shortage and users are unable to handle routine requests and features.

Probe—this attack enumerates the possible flows or defenselessness of the target network that it leverages to initiate further attacks.

Remote to Local (R2L)—invader lacks direct access to the target system, so it attempts to obtain local/remote access to a device of the system.

User to Root (U2R)—an intruder tries to enter the network as a benign user and utilizes the weakness of such system to obtain root access.

### 4.2. Implementation Details

The proposed CFLVAE-DNN was implemented in a Python environment using TensorFlow [73] as backend with Keras [74] higher-level framework on the GPU enabled Google Colaboratory [75] with 12 GB RAM. In our proposed CFLVAE, we used fully connected networks for both the encoder and decoder. Apart from the input and output layers, we defined three hidden layers. Further, we implemented the RELU6 [67,68] activation function to avoid vanishing gradient issues for all hidden layers of encoder and decoder networks. However, Sigmoid is implemented as an activation function for the final layer of the decoder network. The hyper-parameters are defined in Table 1.

The optimal network architecture of the proposed generator CFLVAE network is 87-40-20-10-20-40-87 with two hidden layers for the encoder and two hidden layers for the decoder and a latent space *Z*. The input vector is selected as 87 by utilizing the power of the MI technique. Similarly, the architecture of the DNN network is 87-40-20-10-5 with three hidden layers. Initially, we considered six (6) hidden layers for DNN architecture. After training the model for different hidden layers, we found that the DNN model with three hidden layers obtains better overall detection performance. This makes our DNN classifier model more lightweight to fit into resource-constrained IoT devices. The output layer of the DNN network consists of five neurons as the dataset contains five attack vectors. We proposed novel CFL as the reconstruction objective function and hence, we established the optimal value of hyper-parameter Gamma (γ) and Alpha (α). Initially, the value of γ was set to 0.5; according to Equation (7). After several trials using seven (7) different values, the optimal value of γ in the CFL function was obtained as 1.30.

Thereafter, for both generator and classifier, we used the Adam algorithm with an initial learning rate of 0.001. Adam is adapted as a benchmark optimizer for deep learning research and it fits well into our proposed model. The learning rate is scheduled with a polynomial decay function with decay steps 10 and power of 0.5 to optimize the learning parameters of the optimizer. Many recent deep learning algorithms adapted HeNormal or GlorotNormal initializers for weight initialization. After training our model with both initializers, we finalized GlorotNormal [76] as a weight initializer. Meanwhile, the value of bias regularizer is set to 0.0005 for all layers in both generator (CFLVAE) and classifier (DNN) after several trials. 20% validation data (from training data) is used to monitor for over-fitting during the training process.

We implemented three-fold cross-validation to validate our DNN classifier. We divided the training dataset into three subsets with an equal fraction of every target class of data. During each training procedure of the classifier, one subset holds out for a testing purpose and the rest two subsets are utilized for training the model. By training the DNN classifier three times, each subset of the sample takes part in both training and testing.

**Figure 4:**
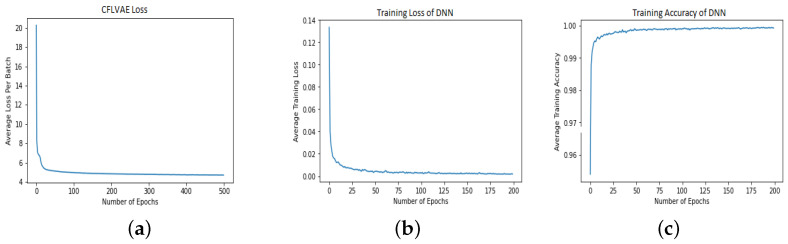
The effects of the training procedure of the CFLVAE and the DNN models. (**a**) CFLVAE loss; (**b**) DNN loss; (**c**) DNN accuracy.

The learning behavior of the CFLVAE and DNN classifier in the proposed CFLVAE-DNN model is depicted in Figure 4. Figure 4a,b presents the loss curves of the CFLVAE data generative model and DNN intrusion classification model respectively. Likewise, Figure 4c shows the accuracy curve for the DNN model. It can be observed that the CFLAVE network converges considerably faster with a minimum number of epochs. The training of the DNN model also reaches high accuracy faster and converges at only 200 epochs.

## 5. Performance of the Proposed CFLVAE-DNN Model

The CFVAE-DNN model has been experimented on a highly imbalanced dataset in a python environment. The following sections present and discuss the intrusion detection performances of CFVAE-DNN and comparative studies.

### 5.1. Data Generation

**Figure 5:**
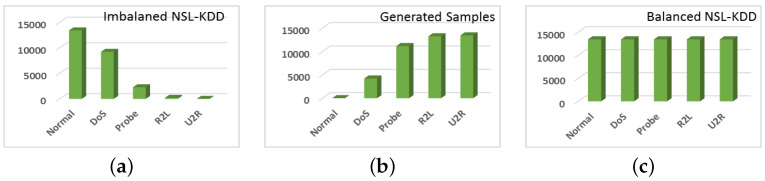
NSL-KDD dataset. (**a**) Imbalanced original records; (**b**) Generated records; (**c**) Balanced dataset.

This section presents the model performance in terms of data generation by the CFLVAE model. The proposed CFLVAE data generation model successfully generates high-quality, diverse, and realistic samples for the minority class attacks. Figure 5a depicts the severely imbalanced NSL-KDD dataset. The generated data is shown in Figure 5b and finally, Figure 5c presents the balanced datasets.

### 5.2. Intrusion Detection

**Figure 6:**
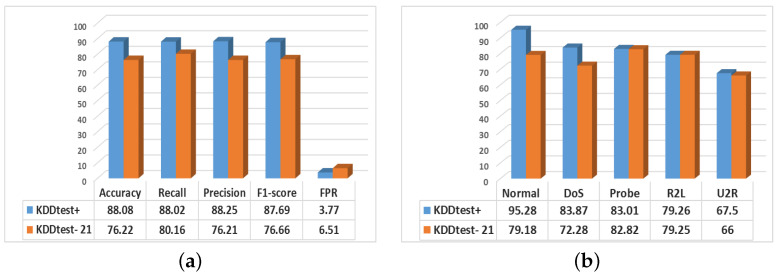
The intrusion detection performance (in %) of our proposed CFLVAE-DNN model. (**a**) Overall performance; (**b**) Class-wise detection rates.

The balanced dataset is used to train the DNN classifier for intrusion detection. Figure 6 presents the overall performance of our proposed CFVAE-DNN model. The overall performance (in %) of our model demonstrated in Figure 6a as follows: accuracy 88.08, recall 88.02, precision 88.25, and F1-score 87.69 are obtained using the KDDtest+ test dataset and similarly, accuracy 76.22, recall 76.21, precision 80.16, and F1-score 76.66 are obtained using the KDDtest-21 test dataset. Moreover, the proposed model achieved significantly low FPR of 3.77% and 6.51% for KDDtest+ and KDDtest-21 test datasets respectively.

Likewise, it is observed that the CFLVAE-DNN improved the overall detection performance of minority attack classes. The class-wise detection scores (in %) are 83.87, 83.01, 79.26, 67.5 for DoS, Probe, R2L and U2R respectively for KDDtest+ dataset and 72.28, 82.82, 79.25, 66.00 for the same minority attacks for KDDtest-21 dataset depicted in Figure 6b.

**Figure 7:**
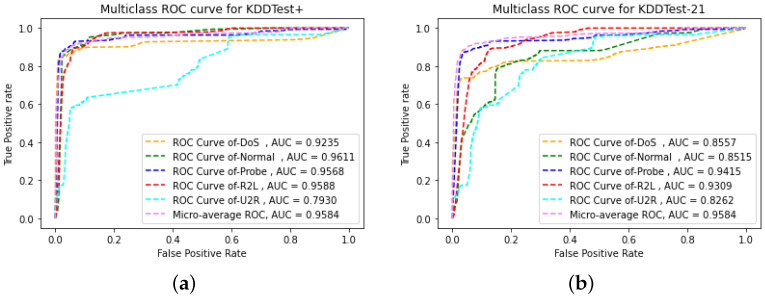
AUC-ROC curve on NSL-KDD test datasets. (**a**) AUC-ROC curve on the KDDTest+; (**b**) AUC-ROC curve on the KDDTest-21.

Consequently, the ROC curves and AUC values are shown in Figure 7. Figure 7a,b present AUC_ROC values for KDDtest+ and KDDtest-21 test datasets respectively. These values play a vital role to analyze the overall performance of learning models. Interestingly, ROC is a graphical representation of FPR on the X-axis versus TPR on the Y-axis, which demonstrates the efficiency of a classification model over diverse threshold values. A higher value of AUC ensures the better performance of the classifier. It is shown in the figures that the AUC values of all classes range between 0.79 and 0.95, which validate that the proposed CFLVAE-DNN generates high-level classification outcome.

#### 5.2.1. Intrusion Detection Using Different DNN Architectures

**Figure 8:**
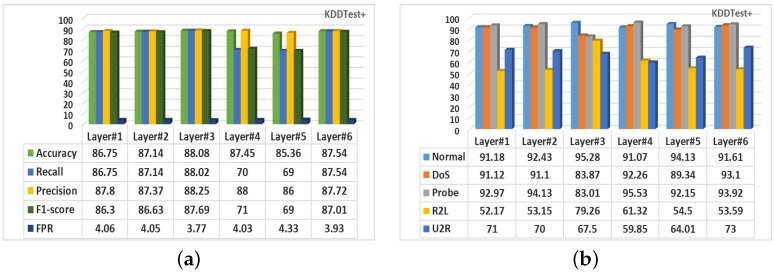
The intrusion detection performance (in %) on different number of hidden layers used in DNN model for KDDTest+ dataset. (**a**) Overall performance; (**b**) Class-wise detection rates.

Furthermore, this research considered several network architectures of the DNN classification model. To make the model suitable for IoT devices, we aim to find the best DNN architecture which has the minimum number of hidden layers and obtains the best intrusion detection performance. The base architecture consists of one input, one output, and six hidden layers. The results of different hidden layers are demonstrated in Figure 8. It is interesting to observe that the intrusion detection performance changes with the different number of hidden layers of the DNN classifier. Consequently, the highest overall detection performance was achieved using three hidden layers, and the lowest overall performance was obtained using five hidden layers on generated data using CFLVAE.

#### 5.2.2. Intrusion Detection Using Different Gamma Values

**Figure 9:**
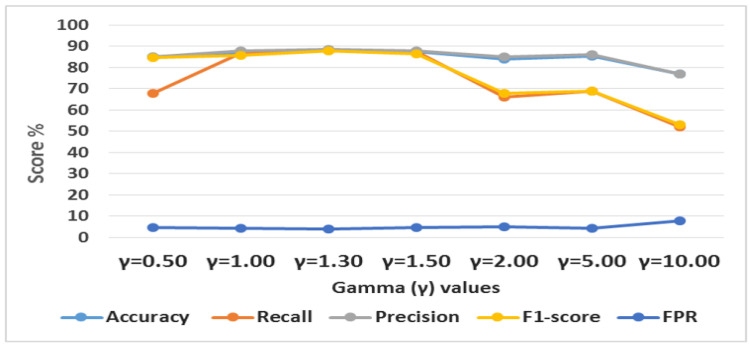
The result of intrusion detection performance with different Gamma (γ) values of Class-wise Focal Loss.

This research proposed a novel CFL loss function as the reconstruction objective function for the CFLVAE model. Additionally, we defined optimal values of Gamma (γ) and Alpha (α) hyper-parameters for the CFL loss function to generate high-quality, diverse, and realistic data samples for low-frequency attacks. The initial value of γ was set to 0.5, according to Equation (7) the optimal value of γ in the CFL function is obtained as 1.30, the value fit for two top minority classes (DoS and Probe) samples, and 1.50 for bottom minority classes (R2L and U2R). This research obtained the (γ) values with trial and error experiments. Figure 9 shows the detection performance of the classifier on different datasets generated using different γ values. The intrusion detection performance is tested using KDDTest-21 dataset. The α value is set to 0.5 for class DoS and Probe and 0.6 for minority class R2L and U2R.

### 5.3. Comparative Study

As mentioned above, the data generation method solves data imbalance issues, which results in improving overall classification accuracy including detection rates of the minority-class attacks. This section provides comparative studies of our proposed model with different existing techniques.

#### 5.3.1. Comparison with Data Generation Methods

Traditionally, Random Over Sampler (ROS) [37], Synthetic Minority Over-sampling Technique (SMOTE) [36], and Adaptive Synthetic (ADASYN) [35] are the most popular oversampling/data generation methods, which have shown significant performance improvement in recent years. Undoubtedly, our proposed CFLVAE-DNN model generates samples for minority and low-frequency attack classes to improve the intrusion detection performance of a deep neural network-based classifier. To compare the overall classification result of the proposed CFLVAE-DNN with the above three most popular data generation methods, we utilized the same DNN model as the classifier.

**Figure 10:**
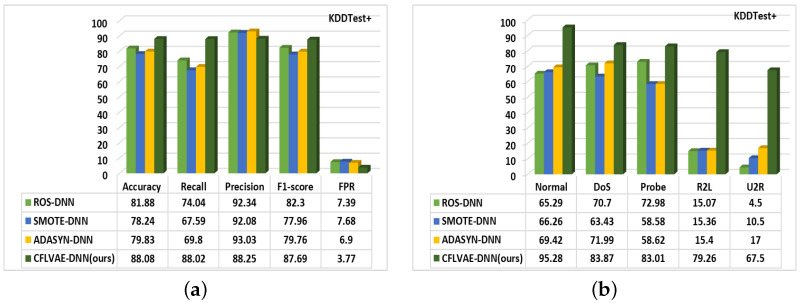
Comparison of (**a**) Overall detection rates and (**b**) Class-wise detection performance of data generation techniques on the KDDTest+ dataset (in %).

**Figure 11:**
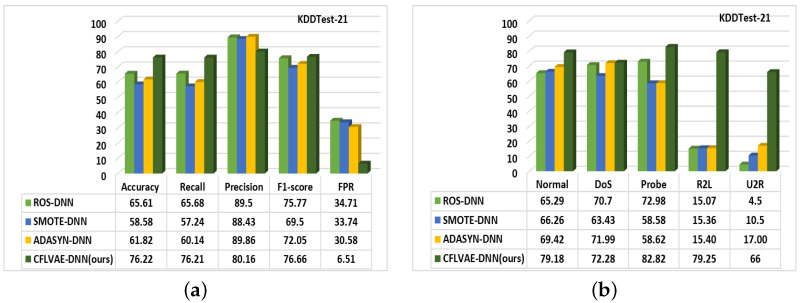
Comparison of (**a**) Overall detection rates and (**b**) Class-wise detection performance of data generation techniques on the KDDTest-21 dataset (in %).

Figure 10 and Figure 11 depict the comparative studies of all three methods with the proposed CFLVAE-DNN. Figure 10a provides overall performance accuracy and Figure 10b provides the class-wise detection performance for the KDDTest+ test dataset. Similarly, Figure 11a provides overall performance accuracy and Figure 11b provides the class-wise detection performance for the KDDTest-21 test dataset. It is interesting to observe that, the CFLVAE-DNN has achieved the highest overall accuracy, recall, precision, and F1-score. The detection rates of minority classes, particularly in R2L (79.26%) and U2R (67.5%) attacks, are the highest among all existing studies.

Meanwhile, our model has also achieved the lowest FPR (e.g., 3.77% & 6.51% for KDDTest+ and KDDTest-21 respectively). These comparative studies demonstrate that the CFLVAE generates more quality and diverse synthetic samples for the minority attack classes. Interestingly, the most significant difference between the mentioned benchmark data generation methods and our proposed CFLVAE is the capability to reconstruct intrusion features from particular attack samples and produce diverse and realistic samples for them. The CFLVAE model can generate a corresponding intrusion sample with its properties. The experimental results confirm that data generated from CFLVAE using class-wise focal loss are more diverse and realistic than the data generated from the benchmark techniques.

The reasons for ROS, SMOTE and ADASYN to perform low detection accuracy may be due to flaws in these techniques. ROS-DNN simply copies the original sample, this could lead to an overfitting problem. SMOTE-DNN uses the KNN algorithm to synthesize samples for minority classes, which is prone to over-generalization. ADASYN-DNN leads to change in the spatial distribution of the observed samples and is subject to outliers.

#### 5.3.2. Comparison with Learning-Based Classifiers

Subsequently, we compare the performance of the proposed CFLVAE-DNN model with seven popular and frequently used ML and DL classifiers, namely, K-Nearest Neighbor (KNN), Gaussian Naive Bayes (GaussianNB), Decision Tree (DT), Random Forest (RF), Support Vector Machine (SVM), Deep Belief Network (DBN), and Deep Neural Network (DNN) [7,8,9,10]. These algorithms are well-established classifiers for their promising performance in intrusion detection and can be found in several literature.

**Figure 12:**
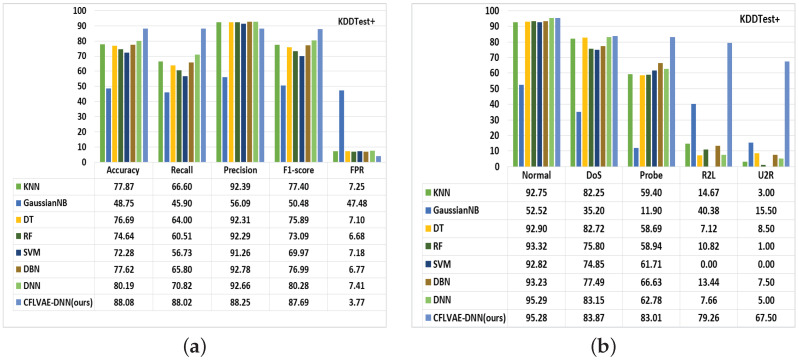
Comparison of (**a**) Overall performance and (**b**) Class-wise detection rates of learning-based classifiers on the NSL-KDD (KDDTest+) dataset (in %).

**Figure 13:**
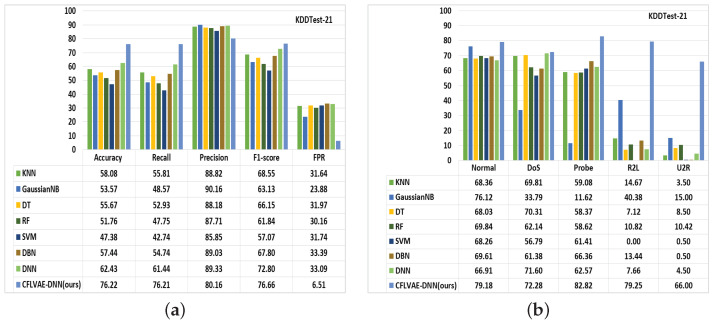
Comparison of (**a**) Overall performance and (**b**) Class-wise detection rates of learning-based classifiers on the NSL-KDD (KDDTest-21) dataset (in %).

The summary of the comparative studies is presented in Figure 12 and Figure 13. As it is depicted from Figure 12a and Figure 13a, the CFLVAE-DNN has a superior detection accuracy (88.08% & 76.22%) and lower FPR (3.77% & 6.51%) among all the well-known classifiers on both NSL-KDDtest+ and NSL-KDDtest-21 test datasets. The figures also demonstrate that the proposed model achieves higher recall and F1-scores. The precision is slightly higher in KNN, SVM, and DBM algorithms.

Furthermore, the CFLVAE-DNN model has achieved higher detection performance for both classes by synthesizing diverse and realistic data for unknown/minority attack types. Figure 12b and Figure 13b, show that CFLVAE-DNN obtains the highest class-wise detection rates for minority attack classes in both NSL-KDDtest+ and NSL-KDDtest-21 datasets. Compared with other detection models, the proposed CFLVAE-DNN obtained the highest detection accuracy (in %) on all minor and significant attack types, namely, DoS (83.87), Probe (83.01), R2L (79.26), and U2R (67.5) NSL-KDDtest+ and DoS (72.28), Probe (82.82), R2L (79.25) and U2R (66.00) NSL-KDDtest-21 test datasets.

#### 5.3.3. Comparison with State-of-the-Art Models

Last but not least, we compared the detection performance of our proposed model with some recently reported intrusion detection techniques to demonstrate the superiority of the CFLVAE-DNN model. The selected state-of-the-art IDS that are reported in the following research: Improved Conditional Variational AutoEncoder (ICVAE-DNN) [60], intrusion detection intrusion detection method based on a Conditional Variational AutoEncoder (ID-CVAE) [53], hybrid IDS called SCDNN [52], Scale-Hybrid-IDS-AlertNet (SHIA) framework [5], Recurrent Neural Network (RNN-IDS) [54], Stacked Non-symmetric Deep AutoEncoders (S-NDAE) [6], and Log-cosh Conditional Variational AutoEncoder (LCVAE) [45].

Table 2 demonstrates the performance comparisons based on the NSL-KDDTest+ test dataset. The majority of the reported state-of-the-art techniques did not consider the NSL-KDDTest-21 test dataset for the evaluation of their models. The comparison is made with regards to the performance matrix. It can be derived from the table that our CFLVAE-DNN obtains the best detection results in terms of F1-score and minority attacks detection rates among all of the intrusion detection models.

Interestingly, our proposed model ranked first in achieving overall F1-score and minority class detection rates. The main aim of CFLVAE-DNN is to improve the minority attacks defection rates, in addition, to improve overall detection performance by solving the data imbalance problem. Our proposed CFLVAE-DNN achieved the highest detection rates for the two rarest unknown attack vectors. Even though the overall accuracy is sightly higher (approx. 3%) in SCDNN [52], the proposed CFLVAE-DNN model obtained the minority attacks class detection rates of 79.26% and 67% against 11.4% and 6.88% achieved by SCDNN for R2L and U2R attacks respectively. It is observed from the table that, by generating a high-quality sample by the proposed CFLVAE model, our DNN algorithm obtains the highest minority attacks detection rates among all other benchmark models.

One of the most important evaluation metrics is F1-score which is the harmonic mean between precision and recall. Although, the precision of our model is negligibly inferior compared to S-NDAE [6], ICVAE-DNN [60] and LCVAE [45] models and the recall is inferior by only about 3% (against SCDNN [52]), the proposed CFLVAE-DNN achieved highest F1-score among all the cited models. Moreover, the ICVAE-DNN [60] scored slightly lower FPR (only 1.03% difference) compared with our CFLVAE-DNN model. However, the ICVAE-DNN model reported inferior detection accuracy, recall, and F1-score, compared to our proposed model. To sum up, the comparative studies demonstrate that the proposed data generation and classification CFLVAE-DNN intrusion detection model is superior in detecting network intrusions including minority attacks effectively.

## 6. Conclusions and Future Work

This paper presented a novel intrusion detection model, which we named CFLVAE-DNN. This study incorporated the strength of Variational AutoEncoder and the effectiveness of Class-wise Focal Loss (CFL) cost-sensitive learning. The first part of the model utilizes the CFL objective function to generate realistic and diverse training samples for specific attack classes to resolve class/data imbalance issues. Consequently, implementing the CFL loss function, the minority-class attack samples receive more attention and the CFLVAE can extract high-level feature distribution of observed samples. The diverse balanced data is then used to train the intrusion classifier, which enables the classifier to achieve higher overall detection performance, higher class-wise detection rates, and lower false-positive rate.

Moreover, we utilized the Deep Neural Network-based classifier with a unique architecture to achieve superior detection performance. Additionally, the most relevant features were selected using the Mutual Information technique to make the model lightweight. Accordingly, the experimental results showed that the proposed CFLVAE-DNN model achieved the highest minority-class attack detection rates (i.e., 79.26% and 67.50% for R2L and U2R respectively) compared to all the benchmark algorithms. Likewise, it also achieved the overall superior intrusion detection performance compared with state-of-the-art data generation-based and traditional learning-based models on NSL-KDD dataset.

The findings presented in this paper are relevant to the deep learning and cyber security community as a whole. Finding a suitable IoT intrusion dataset is a challenge. Considering future research, it will be worthwhile to conduct further investigations on various IoT intrusion datasets. Secondly, although the techniques utilized in this study makes the DNN model lightweight, the implementation of the model in IoT device was out of the scope in this study. Therefore, in future, it will be interesting to study network compression techniques to ensure the suitability of the model for IoT, and, finally, implement the model in a resource-constrained IoT device. Last but not the least, we plan to study different cost functions for data generation technique to alleviate the problem of imbalanced classes to further improve the intrusion detection performance of the CFLVAE-DNN model.

## Figures and Tables

**Table 1:** Hyperparameters.

Hyperparameter		Value
CFLVAE architecture		87-40-20-10-20-40-87
DNN architecture		87-40-20-10-5
Latent space dimension (z)		10
Weight initializer		GlorotNormal
Optimizer		Adam
Learning rate (lr)	Value (lr):	$10^{-3}$ to $10^{-5}$
	Scheduler name:	Polynomial Decay
	Decay step:	10
	Power:	0.5
Focal loss (Gamma value)		0.50, 1.00, 1.30, 1.50, 2.00, 5.00, 10.00
Focal loss (Alpha value)		0.5 and 0.6
Batch size *m*		64
Epochs *ep* (CFLVAE and DNN)		500 and 200

**Table 2:** Comparative study (in %) of CFLVAE-DNN with the state-of-the-art techniques on the KDDTest+ dataset (NA means not available, * ranked first, ** ranked second).

Model	Accuracy	Recall	Precision	F1-Score	FPR	Normal	DoS	Probe	R2L	U2R
ICVAE-DNN [60]	85.97	77.43	97.39	86.27	2.74 *	97.26	85.65	74.97	44.41	11.00
ID-CVAE [53]	80.1	80.1	81.59	79.00	8.18	91.8	84.41	72.78	33.59	0.057
SCDNN [52]	91.97	91.68	NA	NA	8.03	97.21	96.87	80.32	11.4	6.88
SHIA [5]	78.5	78.5	80.1	76.5	NA	97.4	76.6	66.3	67.20	24.20
RNN-IDS [54]	83.28	73.125	NA	83.22	3.44 **	NA	83.49	83.4	24.69	11.5
LCVAE [45]	85.51	68.9	97.61 **	80.78	NA	NA	NA	NA	NA	NA
S-NDAE [6]	85.82	85.82	100 *	87.37	14.58	99.49	99.79	98.74	9.31	NA
**CFLVAE-DNN (ours)**	88.08 **	88.02 **	**88.25**	87.69 *	**3.77**	**95.28**	83.87 **	83.01 **	79.26 *	67.50 *

## Data Availability

The data presented in this study are available in the ref. [55].
